# Consumption of Total and Specific Alcoholic Beverages and Long-Term Risk of Gout Among Men and Women

**DOI:** 10.1001/jamanetworkopen.2024.30700

**Published:** 2024-08-28

**Authors:** Jie-Qiong Lyu, Meng-Yuan Miao, Jia-Min Wang, Yu-Wen Qian, Wen-Wen Han, Xian-Zhen Peng, Hao-Wei Tao, Jing Yang, Jing-Si Chen, Li-Qiang Qin, Wei Chen, Guo-Chong Chen

**Affiliations:** 1 Department of Nutrition and Food Hygiene, School of Public Health, Suzhou Medical College of Soochow University, Suzhou, China; 2 Jiangsu Key Laboratory of Preventive and Translational Medicine for Major Chronic Non-communicable Diseases, Suzhou Medical College of Soochow University, Suzhou, China; 3 MOE Key Laboratory of Geriatric Diseases and Immunology, Suzhou Medical College of Soochow University, Suzhou, China; 4 Department of Public Health, Kangda College of Nanjing Medical University, Lianyungang, China; 5 Department of Clinical Nutrition, The First Affiliated Hospital of Soochow University, Suzhou, China; 6 Department of Clinical Nutrition, Peking Union Medical College Hospital, Chinese Academy of Medical Sciences and Peking Union Medical College, Beijing, China

## Abstract

**Importance:**

Previous studies on alcohol consumption and incident gout have mostly included men or combined both sexes, and the sex-specific associations between alcohol consumption and gout are poorly understood.

**Objective:**

To evaluate the consumption of total and specific alcoholic beverages in association with incident gout in men and women.

**Design, Setting, and Participants:**

This prospective cohort study included 401 128 participants in the UK Biobank aged 37 to 73 years who were free of gout at baseline (2006-2010). Participants were followed up through December 31, 2021, and data were analyzed between August 2023 and June 2024.

**Exposure:**

Questionnaire-based consumption of total alcohol and specific alcoholic beverages.

**Main Outcomes and Measures:**

The outcome was incident gout, identified using hospital records. Multivariable Cox proportional hazards regression models were used to estimate sex-specific hazard ratios (HRs) and 95% CIs of incident gout associated with alcohol consumption, with a particular consideration of reverse causation bias.

**Results:**

The main analysis included 179 828 men (mean [SD] age, 56.0 [8.2] years) and 221 300 women (mean [SD] age, 56.0 [8.0] years). Current drinkers showed a higher risk of gout than never drinkers among men (HR, 1.69; 95% CI, 1.30-2.18) but not among women (HR, 0.83; 95% CI, 0.67-1.03). Among current drinkers, higher total alcohol consumption was associated with a higher risk of gout among both sexes and more strongly among men than women (men: HR, 2.05 [95% CI, 1.84-2.30]; women: HR, 1.34 [95% CI, 1.12-1.61]). The most evident sex difference in the consumption of specific alcoholic beverages was observed for beer or cider (men: mean [SD], 4.2 [4.8] pints per week; women: mean [SD], 0.4 [1.1] pints per week). Consumption of champagne or white wine, beer or cider, and spirits each was associated with a higher risk of gout among both sexes, with beer or cider showing the strongest association per 1 pint per day (men: HR, 1.60 [95% CI, 1.53-1.67]; women: HR, 1.62 [95% CI, 1.02-2.57]). Some inverse associations between light to moderate consumption of specific alcoholic beverages and gout were eliminated after adjusting for other alcoholic beverages and excluding individuals who had reduced alcohol consumption for health reasons, self-reported poor health, or had cardiovascular disease, cancer, or kidney failure at baseline, or developed gout within the first 2 years of follow-up.

**Conclusions and Relevance:**

In this cohort study, higher consumption of several specific alcoholic beverages was associated with a higher risk of gout among both sexes. The sex-specific associations for total alcohol consumption may be associated with differences between men and women in the types of alcohol consumed.

## Key Points

**Question:**

Is the association between alcohol consumption and risk of gout sex specific?

**Findings:**

In this cohort study involving 401 128 participants from the UK Biobank, higher total alcohol consumption was associated with a higher risk of gout for both sexes, more strongly for men than for women. Consumption of several specific alcoholic beverages (especially beer or cider) was associated with a higher risk of gout, similarly for men and women.

**Meaning:**

These findings suggest that specific alcoholic beverages are associated with a similar risk of gout for men and women and that alcohol consumption should be minimized for gout prevention regardless of sex.

## Teaser

This cohort study of more than 400 000 participants from the UK Biobank evaluates whether there is a sex-specific difference in the association of consumption of total and specific alcoholic beverages with incident gout.

## Introduction

Gout, which develops in the presence of increased urate concentrations,^1^ is the most common form of inflammatory arthritis.^2^ The prevalence of gout is sex specific and varies across global regions, with a male to female ratio ranging from 2:1 to 4:1 in Europe and North America and a substantially higher ratio of approximately 8:1 in Asia.^2^

A sustained elevation in serum urate levels is the main factor associated with the development of gout.^2^ Although genetic susceptibility plays an important role,^2,3^ lifestyle factors are also involved in the development of hyperuricemia and gout.^2,4^ Specifically, alcohol intake has been associated with elevated serum urate levels^5^ and thus might eventually cause gout through hyperuricemia.^6^ A number of epidemiologic studies have suggested total or specific alcohol intake to be associated with a higher risk of gout.^6,7,8,9,10,11,12,13,14,15^ These studies were limited by using a cross-sectional or case-control design^8,10,14,15^ or by including only men.^6,9,10,13^ In a cohort study comprising 1951 men and 2476 women, consumption of beer or spirits was associated with a higher risk of gout more strongly among women than men,^7^ indicating possible sex-specific associations.

Furthermore, the previous studies of alcohol consumption and incident gout have commonly used nondrinkers as the referent population, such that the effect of reverse causation bias on the examined association remains an open issue. It has been found that, when evaluating the connection between lifestyle factors and health outcomes, reverse causation can influence the magnitude and sometimes the direction of the examined association.^16^ In the case of alcohol consumption, individuals with ill health may have abstained from alcohol, shifting into nondrinking or occasional drinking categories,^17^ which may attenuate or reverse a positive association, or exaggerate an inverse association between alcohol consumption and health risk. Such a reverse causation bias has been acknowledged as a factor associated with the widely observed U- or J-shaped association between alcohol consumption and risk of cardiovascular disease or premature mortality.^17,18^ U-shaped associations also have been observed between serum uric acid levels and mortality.^19,20,21^

To address these research gaps and to gain more accurate risk estimates, we conducted sex-specific analyses to investigate the associations of total and specific alcohol consumption with the long-term risk of gout, with a particular consideration of addressing potential reverse causation bias.

## Methods

### Study Design and Population

The UK Biobank constitutes a vast prospective cohort investigation aiming to explore the fundamental determinants of diverse chronic conditions, encompassing genetic, environmental, and lifestyle factors.^22^ Between 2006 and 2010, the study recruited approximately 500 000 men and women aged between 37 and 73 years. Enrolled participants were invited to visit 1 of the 22 assessment centers situated across England, Wales, and Scotland, where they underwent extensive questionnaire surveys, brief interviews, and physical assessments. Participants were followed up through December 31, 2021. The UK Biobank study received approval from the UK North West Multicentre Research Ethics Committee, and all participants provided informed consent at enrollment. This study followed the Strengthening the Reporting of Observational Studies in Epidemiology (STROBE) reporting guideline for cohort studies.

### Assessment of Alcohol Consumption

Information on alcohol consumption at baseline was obtained using a computer-assisted touchscreen system. Participants were asked to report the status of alcohol drinking as never, previous, or current drinking. Current drinkers were asked to respond to additional questions regarding how much of each alcoholic beverage (red wine, champagne or white wine, beer or cider [ie, bitter, lager, stout, ale, and Guinness, all of which contain alcohol], spirits, and fortified wine) they consumed in an average week (or month for those who drank less than once per week).^23^ Weekly alcohol consumption was categorized into 4 groups (<1, 1-2, 3-4, and ≥5 times per week). Specific alcohol consumption was categorized into 5 groups (0, ≤1, 2-3, 4-6, and ≥7 glasses of red wine, glasses of champagne or white wine, pints of beer or cider, measures of spirits, or glasses of fortified wine per week). To account for variations in alcohol content and drink volumes across beverages, we also estimated the amount of ethanol from specific alcoholic beverages (in grams per day) using previously reported methods.^24^

### Assessment of Gout

In line with the previous analyses of data from the UK Biobank,^25,26,27^ participants with gout at baseline were identified based on self-reported data, including a self-reported history of gout and use of urate-lowering therapy (allopurinol, probenecid, and/or sulfinpyrazone; febuxostat was not present in the database) or colchicine, in addition to a hospital diagnosis of gout. Incident gout during follow-up was determined from hospital diagnoses recorded in primary or secondary inpatient records, using the *International Classification of Diseases, Ninth Revision* (codes 274.0, 274.1, 272.8, and 274.9) and the *International Statistical Classification of Diseases and Related Health Problems, Tenth Revision* (codes M10.0, M10.2, M10.3, M10.4, and M10.9).

### Assessment of Covariates

Information on sociodemographic factors, medical histories and medication use (including diuretic use), and lifestyle behaviors (eg, smoking, usual physical activity, and dietary habits over the past year) was collected at baseline by nurse-led interviews and touchscreen questionnaires.^28,29^ Race and ethnicity were assessed due to the possibility of different racial and ethnic backgrounds being associated with the incidence of gout. Race and ethnicity were self-reported and included Asian or Asian British, Black or Black British, White, and other (ie, Asian and White, Black and White African, Black and White Caribbean, or other multiethnic groups). Anthropometric and blood pressure measurements were performed by trained staff following standard procedures. The Townsend Deprivation Index was derived by combining 4 census variables (unemployment, noncar ownership, nonhome ownership, and household overcrowding). Body mass index (BMI) was calculated using measured weight and height (calculated as weight in kilograms divided by height in meters squared). The estimated glomerular filtration rate was calculated according to the Chronic Kidney Disease Epidemiology Collaboration equation.^30^ Baseline diabetes, hypertension, dyslipidemia, and other chronic diseases were defined appropriately as reported elsewhere.^28^

### Exclusion Criteria

**Figure 1.**
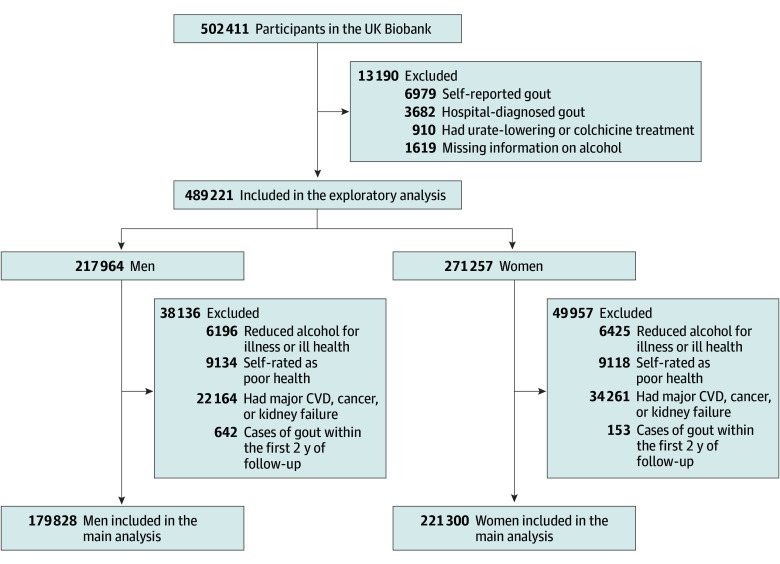
Flow Diagram of Participant Selection CVD indicates cardiovascular disease.

As shown in Figure 1, sample sizes varied across different analyses. In the exploratory analysis, we excluded participants with self-reported gout, hospital-diagnosed gout, urate-lowering treatment, or missing information on drinking at baseline. To mitigate potential reverse causation and perform the main analysis, we further excluded participants who (1) had reduced alcohol intake for illness or ill health at baseline; (2) self-rated as having poor health at baseline; (3) had major cardiovascular disease (ie, coronary heart disease, stroke, or heart failure), cancer (except nonmelanoma skin cancer), or kidney failure at baseline; and (4) developed gout within the first 2 years of follow-up (ie, to minimize the inclusion of prevalent cases undiagnosed at baseline).

### Statistical Analysis

Statistical analysis was conducted between August 2023 and June 2024. Baseline participant characteristics were summarized based on status (for all participants) or frequency (for current drinkers) of total alcohol drinking among men and women. Data were reported as number, percentage, or mean (SD) value where appropriate. Missing data for continuous covariates were addressed using sex-specific median values, and categorical covariates were addressed with a missing indicator category. Correlations among the specific alcoholic beverages were assessed using the Spearman partial correlation coefficients.

Cox proportional hazards regression models were used to estimate sex-specific hazard ratios (HRs) and 95% CIs for the association of total or specific alcohol consumption with incident gout. The proportional hazards assumptions of the models were satisfied as indicated by Kaplan-Meier curves (eFigure 1 in Supplement 1) and the Schoenfeld residual method (*P* > .40). Follow-up time was calculated from the date of enrollment through the date of diagnosis of gout, death, withdrawal from the study, or the end of the most recent follow-up for the approved dataset, whichever occurred first. Different multivariable models with an increasing degree of covariate adjustment were constructed. Model 1 was adjusted for age, racial and ethnic background, Townsend Deprivation Index, smoking, total physical activity, hypertension, dyslipidemia, diabetes, estimated glomerular filtration rate, diuretic use, and major food groups (ie, red meat, processed meat, poultry, oily fish, nonoily fish, fresh fruit, and fresh vegetables). Model 2 was further adjusted for BMI. For the analysis on a specific alcoholic beverage, we used an additional model (model 3) with further adjustment for other types of alcoholic beverages. We also modeled alcohol intake as a continuous variable (each increment of 1 serving/week) and estimated the corresponding HRs and 95% CIs of gout. In addition, we evaluated the amount of daily ethanol (8 g/d, which represents ethanol from 1 standard drink in the UK^31^) from total or specific alcohol in association with incident gout among current drinkers.

To address potential reverse causation bias, the main analysis was performed using the full models after excluding the aforementioned 4 groups of participants, both 1 by 1 and concurrently. All *P* values were from 2-sided tests and results were deemed statistically significant at *P* < .05. Statistical analysis was performed using Stata, version 15.1 (StataCorp), and R, version 4.2.2 (R Project for Statistical Computing).

## Results

### Baseline Participant Characteristics

There were 217 964 men and 271 257 women included for the exploratory analysis. Among these, 179 828 men (mean [SD] age, 56.0 [8.2] years; 2.3% Asian or Asian British participants, 1.5% Black or Black British participants, 95.7% White participants, and 0.5% participants of other race or ethnicity) and 221 300 women (mean [SD] age, 56.0 [8.0] years; 1.7% Asian or Asian British participants, 1.7% Black or Black British participants, 95.9% White participants, and 0.7% participants of other race or ethnicity) were included in the main analysis (Figure 1). Among men, 6253 (2.9%) were never drinkers, 7792 (3.6%) were former drinkers, and 203 919 (93.6%) were current drinkers. Among women, 15 879 (5.9%) were never drinkers, 9881 (3.6%) were former drinkers, and 245 497 (90.5%) were current drinkers. As shown in eTable 1 in Supplement 1, regardless of sex, compared with never drinkers, current drinkers were more likely to be White, were less likely to be never smokers or have dyslipidemia or diabetes, and had lower levels of the Townsend Deprivation Index (higher socioeconomic status) and BMI. Some participant characteristics (eg, age and hypertension) also showed sex-specific distributions according to drinking status. Sex-specific participant characteristics according to the frequency of alcohol consumption among current drinkers are reported in eTable 2 in Supplement 1.

### Alcohol Consumption and Risk of Gout Among Men and Women

Among the participants in the exploratory analysis, 8639 incident cases of gout (6561 cases in men and 2078 cases in women) were identified over a median follow-up time of 12.7 years (IQR, 12.1-13.5 years). The number of incident cases of gout was 5278 (4096 in men and 1182 in women) in the main analysis.

Among men, compared with never drinkers, current drinkers had a higher risk of gout in the exploratory analysis with multivariable adjustment (model 2; HR, 1.57; 95% CI, 1.31-1.89), and the association became stronger in the main analysis accounting for potential reverse causation (HR, 1.69; 95% CI, 1.30-2.18) (eTable 3 in Supplement 1). Among women, the association was the inverse but not significant in the main analysis (HR, 0.83; 95% CI, 0.67-1.03), with significant interaction between drinking status and sex (*P* < .001 for interaction).

We then evaluated the frequency of alcohol consumption and risk of gout among current drinkers (Table 1). Among male drinkers, the risk of gout increased with increasing alcohol intake frequency, with an HR of 2.05 (95% CI, 1.84-2.30) in the main analysis comparing 5 or more times per week with less than 1 time per week of alcohol consumption. Among female drinkers, a positive association was present only when BMI was added to the multivariable model and became stronger after accounting for reverse causation, with an HR of 1.34 (95% CI, 1.12-1.61) comparing 5 or more times per week with less than 1 time per week of alcohol consumption in the main analysis.

**Table 1.** Frequency of Alcohol Consumption and Incident Gout Among Current Drinkers

Model	Risk of gout, HR (95% CI)				*P* value for trend
	<1 Drink/wk	1-2 Drinks/wk	3-4 Drinks/wk	≥5 Drinks/wk	
**Men**					
No. of cases of gout/total No. of participants	767/36 110	1543/56 773	1807/56 672	2123/54 364	NA
Model 1^a^	1.00 [Reference]	1.35 (1.24-1.48)	1.59 (1.46-1.73)	1.85 (1.70-2.01)	<.001
Model 2^b^	1.00 [Reference]	1.41 (1.29-1.54)	1.74 (1.59-1.90)	2.10 (1.92-2.28)	<.001
Model 2^c^	1.00 [Reference]	1.41 (1.28-1.54)	1.76 (1.61-1.93)	2.11 (1.93-2.31)	<.001
Model 2^d^	1.00 [Reference]	1.42 (1.30-1.56)	1.75 (1.59-1.92)	2.12 (1.94-2.33)	<.001
Model 2^e^	1.00 [Reference]	1.40 (1.26-1.55)	1.78 (1.61-1.97)	2.15 (1.95-2.38)	<.001
Model 2^f^	1.00 [Reference]	1.38 (1.25-1.51)	1.66 (1.51-1.82)	2.04 (1.86-2.23)	<.001
Model 2^g^	1.00 [Reference]	1.34 (1.19-1.50)	1.68 (1.50-1.88)	2.05 (1.84-2.30)	<.001
**Women**					
No. of cases of gout/total No. of participants	682/76 249	448/69 881	327/55 693	315/43 674	NA
Model 1^a^	1.00 [Reference]	0.88 (0.77-0.99)	0.86 (0.75-0.98)	0.94 (0.82-1.08)	.18
Model 2^b^	1.00 [Reference]	0.98 (0.87-1.11)	1.04 (0.91-1.20)	1.21 (1.05-1.39)	.02
Model 2^c^	1.00 [Reference]	0.99 (0.88-1.13)	1.07 (0.93-1.24)	1.25 (1.08-1.45)	.003
Model 2^d^	1.00 [Reference]	1.01 (0.89-1.15)	1.08 (0.93-1.24)	1.26 (1.08-1.46)	.004
Model 2^e^	1.00 [Reference]	0.97 (0.84-1.12)	1.01 (0.86-1.18)	1.24 (1.05-1.46)	.03
Model 2^f^	1.00 [Reference]	0.99 (0.87-1.13)	1.03 (0.89-1.20)	1.21 (1.04-1.41)	.02
Model 2^g^	1.00 [Reference]	1.02 (0.87-1.20)	1.06 (0.88-1.26)	1.34 (1.12-1.61)	.004

Abbreviations: HR, hazard ratio; NA, not applicable.

^a^ Model 1 was adjusted for age, racial and ethnic group (Asian or Asian British, Black or Black British, White, or other), Townsend Deprivation Index, smoking (never, former, or current [<10, 10 to <50, or ≥50 pack-years]), total physical activity (metabolic equivalent of task–hour/week), hypertension (yes or no), dyslipidemia (yes or no), diabetes (yes or no), estimated glomerular filtration rate, diuretic use (yes or no), and major food groups (red meat, processed meat, poultry, oily fish, nonoily fish, fresh fruit, and fresh vegetables).

^b^ Model 2 was additionally adjusted for body mass index.

^c^ Excluding participants who had reduced alcohol intake for illness or ill health at baseline.

^d^ Excluding participants self-rating as having poor health at baseline.

^e^ Excluding participants with major cardiovascular disease, cancer, or kidney failure at baseline.

^f^ Excluding cases of gout occurring within the first 2 years of follow-up.

^g^ Excluding participants who had reduced alcohol intake for illness or ill health at baseline; self-rated as having poor health at baseline; had major cardiovascular disease, cancer, or kidney failure at baseline; and developed gout within the first 2 years of follow-up.

### Consumption of Specific Alcoholic Beverages and Risk of Gout Among Men and Women

**Figure 2.**
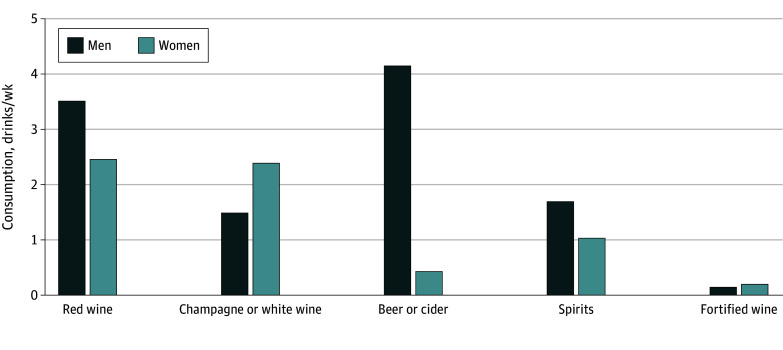
Weekly Mean Consumption of Alcoholic Beverages Among Male and Female Current Drinkers

The correlations among the specific alcoholic beverages were modest (eFigure 2 in Supplement 1). Consumption of different alcoholic beverages varied between sexes, and the most evident difference was observed for beer or cider, with a mean (SD) of 4.2 (4.8) pints per week consumed by men and 0.4 pints (1.1) per week consumed by women (Figure 2).

We then investigated the associations between different alcoholic beverages and incident gout among men (Table 2) and women (Table 3). In the main analysis, consumption of champagne or white wine, beer or cider, and spirits each was associated with a higher risk of gout among both sexes, with beer or cider showing the strongest association per 1 pint per day (men: HR, 1.60; 95% CI, 1.53-1.67 [ Table 2]; women: HR, 1.62; 95% CI, 1.02-2.57 [ Table 3]). The association for spirits per 1 measure per day appeared to be stronger among women (HR, 1.54; 95% CI, 1.29-1.83 [Table 3]) than men (HR, 1.12; 95% CI, 1.06-1.19 [Table 2]). Red wine per 1 glass per day was associated with a modestly higher risk of gout among men (HR, 1.13; 95% CI, 1.07-1.19 [Table 2]) but not among women. No significant association was found for fortified wine for either sex.

**Table 2.** Consumption of Specific Alcoholic Beverages and Incident Gout Among Male Current Drinkers

Alcoholic beverage	Risk of gout, HR (95% CI)					*P* value for trend	Per 1 serving/d, HR (95% CI)
	0 Drinks/wk	≤1 Drink/wk	2-3 Drinks/wk	4-6 Drinks/wk	≥7 Drinks/wk		
**Red wine, glasses/wk**							
No. of cases of gout/total No. of participants	2105/61 413	467/17 674	954/32 888	943/32 781	979/28 804	NA	NA
Model 2^a^	1.00 [Reference]	0.85 (0.77-0.94)	0.92 (0.85-0.99)	0.92 (0.85-1.00)	1.05 (0.97-1.13)	.02	1.05 (1.01-1.09)
Model 3^b^	1.00 [Reference]	1.06 (0.95-1.19)	1.08 (0.99-1.18)	1.05 (0.96-1.15)	1.24 (1.14-1.35)	<.001	1.12 (1.07-1.17)
Model 3^c^	1.00 [Reference]	1.01 (0.88-1.17)	1.03 (0.92-1.15)	1.00 (0.90-1.12)	1.23 (1.11-1.37)	<.001	1.13 (1.07-1.19)
**Champagne or white wine, glasses/wk**							
No. of cases of gout/total No. of participants	3308/99 604	585/22 341	810/29 016	521/16 648	304/8328	NA	NA
Model 2^a^	1.00 [Reference]	0.86 (0.79-0.94)	0.90 (0.83-0.98)	1.01 (0.92-1.11)	1.18 (1.05-1.33)	.003	1.11 (1.04-1.19)
Model 3^b^	1.00 [Reference]	1.03 (0.93-1.13)	0.98 (0.90-1.06)	1.08 (0.97-1.19)	1.32 (1.16-1.50)	<.001	1.17 (1.08-1.25)
Model 3^c^	1.00 [Reference]	1.07 (0.94-1.21)	1.02 (0.92-1.13)	1.13 (1.00-1.28)	1.47 (1.27-1.70)	<.001	1.24 (1.14-1.36)
**Beer or cider, pints/wk**							
No. of cases of gout/total No. of participants	941/37 449	509/26 934	1003/37 443	1103/32 985	1806/37 721	NA	NA
Model 2^a^	1.00 [Reference]	0.81 (0.73-0.89)	1.13 (1.04-1.24)	1.42 (1.30-1.55)	2.05 (1.89-2.22)	<.001	1.52 (1.47-1.57)
Model 3^b^	1.00 [Reference]	0.83 (0.74-0.94)	1.17 (1.06-1.29)	1.49 (1.36-1.64)	2.25 (2.06-2.46)	<.001	1.58 (1.53-1.64)
Model 3^c^	1.00 [Reference]	0.91 (0.79-1.05)	1.18 (1.04-1.33)	1.48 (1.31-1.67)	2.36 (2.11-2.64)	<.001	1.60 (1.53-1.67)
**Spirits, measures/wk**							
No. of cases of gout/total No. of participants	3307/109 263	463/18 225	744/22 665	510/14 753	550/12 089	NA	NA
Model 2^a^	1.00 [Reference]	0.81 (0.73-0.89)	0.97 (0.89-1.05)	0.95 (0.86-1.04)	1.16 (1.06-1.27)	<.001	1.08 (1.04-1.13)
Model 3^b^	1.00 [Reference]	0.94 (0.85-1.05)	1.00 (0.92-1.09)	0.92 (0.84-1.02)	1.17 (1.06-1.29)	<.001	1.09 (1.04-1.14)
Model 3^c^	1.00 [Reference]	1.00 (0.88-1.14)	1.00 (0.90-1.11)	0.93 (0.82-1.06)	1.27 (1.13-1.44)	<.001	1.12 (1.06-1.19)
**Fortified wine, glasses/wk**							
No. of cases of gout/total No. of participants	5241/164 631	217/8474	132/4420	62/1430	10/386	NA	NA
Model 2^a^	1.00 [Reference]	0.83 (0.73-0.95)	0.89 (0.75-1.06)	1.23 (0.95-1.58)	0.75 (0.40-1.40)	.44	0.90 (0.69-1.17)
Model 3^b^	1.00 [Reference]	0.95 (0.82-1.10)	0.94 (0.78-1.14)	1.37 (1.06-1.78)	0.86 (0.45-1.66)	.73	1.05 (0.80-1.39)
Model 3^c^	1.00 [Reference]	0.99 (0.83-1.18)	0.98 (0.78-1.23)	1.46 (1.06-2.01)	1.39 (0.72-2.68)	.16	1.26 (0.91-1.75)

Abbreviations: HR, hazard ratio; NA, not applicable.

^a^ Model 2 was adjusted for age, racial and ethnic group (Asian or Asian British, Black or Black British, White, or other), Townsend Deprivation Index, smoking (never, former, or current [<10, 10 to <50, or ≥50 pack-years]), total physical activity (metabolic equivalent of task–hour/week), hypertension (yes or no), dyslipidemia (yes or no), diabetes (yes or no), estimated glomerular filtration rate, diuretic use (yes or no), major food groups (red meat, processed meat, poultry, oily fish, nonoily fish, fresh fruit, and fresh vegetables), and body mass index.

^b^ Model 3 was additionally adjusted for other types of alcoholic beverages.

^c^ Excluding participants who had reduced alcohol intake for illness or ill health at baseline; self-rated as having poor health at baseline; had major cardiovascular disease, cancer, or kidney failure at baseline; and developed gout within the first 2 years of follow-up.

**Table 3.** Consumption of Specific Alcoholic Beverages and Incident Gout Among Female Current Drinkers

Alcoholic beverage	Risk of gout, HR (95% CI)					*P* value for trend	Per 1 serving/d, HR (95% CI)
	0 Drinks/wk	≤1 Drink/wk	2-3 Drinks/wk	4-6 Drinks/wk	≥7 Drinks/wk		
**Red wine, glasses/wk**							
No. of cases of gout/total No. of participants	622/75 053	148/23 862	221/38 014	179/31 906	99/19 085	NA	NA
Model 2^a^	1.00 [Reference]	0.82 (0.69-0.99)	0.85 (0.72-0.99)	0.83 (0.70-0.99)	0.77 (0.62-0.95)	.004	0.82 (0.72-0.94)
Model 3^b^	1.00 [Reference]	0.97 (0.79-1.18)	0.94 (0.80-1.11)	0.91 (0.76-1.09)	0.82 (0.66-1.03)	.06	0.87 (0.76-1.00)
Model 3^c^	1.00 [Reference]	0.82 (0.62-1.09)	1.02 (0.83-1.26)	0.94 (0.75-1.19)	0.87 (0.65-1.15)	.37	0.92 (0.77-1.10)
**Champagne or white wine, glasses/wk**							
No. of cases of gout/total No. of participants	551/72 888	169/29 669	238/38 166	158/29 003	119/17 876	NA	NA
Model 2^a^	1.00 [Reference]	0.79 (0.66-0.94)	0.97 (0.83-1.13)	0.93 (0.78-1.11)	1.20 (0.98-1.46)	.12	1.10 (0.97-1.25)
Model 3^b^	1.00 [Reference]	0.89 (0.73-1.08)	1.01 (0.86-1.19)	0.97 (0.80-1.17)	1.26 (1.02-1.56)	.05	1.14 (1.00-1.30)
Model 3^c^	1.00 [Reference]	0.82 (0.63-1.06)	0.97 (0.79-1.21)	1.03 (0.82-1.31)	1.31 (1.00-1.71)	.04	1.19 (1.01-1.40)
**Beer or cider, pints/wk**							
No. of cases of gout/total No. of participants	1027/149 642	130/24 144	79/12 793	41/5460	15/1104	NA	NA
Model 2^a^	1.00 [Reference]	0.88 (0.74-1.06)	1.10 (0.87-1.39)	1.22 (0.89-1.67)	2.38 (1.42-3.97)	.006	1.58 (1.14-2.18)
Model 3^b^	1.00 [Reference]	0.94 (0.77-1.14)	1.07 (0.84-1.38)	1.29 (0.93-1.79)	2.36 (1.36-4.10)	.002	1.71 (1.22-2.40)
Model 3^c^	1.00 [Reference]	0.84 (0.64-1.10)	0.99 (0.71-1.38)	1.15 (0.73-1.84)	2.81 (1.44-5.48)	.04	1.62 (1.02-2.57)
**Spirits, measures/wk**							
No. of cases of gout/total No. of participants	746/129 492	130/20 893	166/21 294	128/12 251	91/7211	NA	NA
Model 2^a^	1.00 [Reference]	0.94 (0.78-1.13)	1.14 (0.96-1.35)	1.42 (1.18-1.72)	1.56 (1.25-1.95)	<.001	1.43 (1.26-1.63)
Model 3^b^	1.00 [Reference]	1.00 (0.82-1.23)	1.16 (0.97-1.38)	1.32 (1.08-1.63)	1.62 (1.29-2.05)	<.001	1.47 (1.28-1.68)
Model 3^c^	1.00 [Reference]	1.11 (0.86-1.44)	1.25 (1.00-1.57)	1.39 (1.07-1.82)	1.74 (1.29-2.34)	<.001	1.54 (1.29-1.83)
**Fortified wine, glasses/wk**							
No. of cases of gout/total No. of participants	1153/173 395	63/11 957	65/6527	15/2269	8/588	NA	NA
Model 2^a^	1.00 [Reference]	0.72 (0.56-0.93)	1.29 (1.00-1.66)	0.85 (0.51-1.42)	1.74 (0.86-3.48)	.44	1.19 (0.77-1.84)
Model 3^b^	1.00 [Reference]	0.73 (0.55-0.96)	1.22 (0.92-1.60)	0.87 (0.51-1.48)	1.42 (0.64-3.17)	.84	1.05 (0.65-1.70)
Model 3^c^	1.00 [Reference]	0.84 (0.60-1.18)	1.10 (0.76-1.60)	0.88 (0.45-1.70)	1.97 (0.81-4.76)	.62	1.17 (0.64-2.11)

Abbreviations: HR, hazard ratio; NA, not applicable.

^a^ Model 2 was adjusted for age, racial and ethnic group (Asian or Asian British, Black or Black British, White, or other), Townsend Deprivation Index, smoking (never, former, or current [<10, 10 to <50, or ≥50 pack-years]), total physical activity (metabolic equivalent of task–hour/week), hypertension (yes or no), dyslipidemia (yes or no), diabetes (yes or no), estimated glomerular filtration rate, diuretic use (yes or no), major food groups (red meat, processed meat, poultry, oily fish, nonoily fish, fresh fruit, and fresh vegetables), and body mass index.

^b^ Model 3 was additionally adjusted for other types of alcoholic beverages.

^c^ Excluding participants who had reduced alcohol intake for illness or ill health at baseline; self-rated as having poor health at baseline; had major cardiovascular disease, cancer, or kidney failure at baseline; and developed gout within the first 2 years of follow-up.

In the exploratory analysis with multivariable adjustment (model 2), light (≤1 serving/week) or moderate (2-3 servings/week) consumption of several specific alcoholic beverages was significantly associated with a lower risk of gout. All these inverse associations were attenuated to be null in the main analysis in which the specific alcoholic beverages were mutually adjusted for each other and the potential reverse causation was further addressed (Table 2 and Table 3). For example, per 1 glass per day, women with any level of red wine intake had a lower risk of gout in the exploratory analysis (HR, 0.82; 95% CI, 0.72-0.94), but not in the main analysis (HR, 0.92; 95% CI, 0.77-1.10 [Table 3]). Results were similar in the analyses assessing the amount of ethanol from total or specific alcohol in association with incident gout (eFigure 3 in Supplement 1).

## Discussion

In this large population-based prospective study, we have assessed the consumption of total alcohol and specific alcoholic beverages in association with incident gout. We first observed sex-specific associations between drinking status and risk of gout, with current drinkers having a higher risk than never drinkers among men but not women. Among current drinkers, after addressing potential confounding and reverse causation, more frequent alcohol consumption was associated with a substantially elevated risk of gout among men and a moderately elevated risk among women. Regardless of sex, greater consumption of several specific alcoholic beverages, especially beer or cider, was associated with a higher risk of gout.

Although a number of studies have linked alcohol consumption with a higher risk of gout,^32^ most have included only men or only women without assessing sex-specific associations. In a community-based Japanese cohort, higher alcohol consumption (vs never drinking) was associated with a higher risk of a composite outcome of hyperuricemia and gout among men, with no association among women.^11^ In the present study, current drinking (vs never drinking) was associated with a higher risk of gout among men only after further adjusting for BMI. Among women, current drinkers showed a lower risk of gout than never drinkers after multivariable adjustment, but this association was absent after further adjusting for BMI. Among current drinkers, men who drank 5 times or more per week had an approximately 2-fold higher risk of gout than those who drank less than once per week. Among female drinkers, a positive association between drinking frequency and incident gout was present only after adjusting for BMI, especially after further accounting for potential reverse causation. For participants (especially women) in the present study, current drinkers and especially frequent drinkers had a lower BMI than never drinkers and occasional drinkers. Thus, a lack of BMI adjustment may offset a detrimental association or even lead to a false inverse association between alcohol consumption and risk of gout.

The observed sex-specific difference in the association of drinking status or frequency with incident gout may be, at least in part, owing to the difference between men and women in the type of alcohol consumed. In particular, women as compared with men consumed a considerably lower amount of beer or cider, which was most strongly associated with gout among the specific alcoholic beverages. On the other hand, gout typically manifests at an older age among women (mainly after menopause) than among men,^33^ leading to a shorter duration of exposure to hyperuricemia and urate crystal deposition among women. Thus, it is unclear whether such sex-specific pathophysiological mechanisms of gout may have been associated with the observed sex difference in the gout risk associated with alcohol consumption.

To our knowledge, only a few studies have assessed the consumption of specific alcoholic beverages in association with risk of gout, and the findings are inconsistent. A prospective study of US male professionals^6^ and the Framingham Heart Study^7^ both found no correlation between wine consumption and gout, whereas in 2 other prospective studies,^12,13^ wine consumption was associated with a higher risk of gout. In the current analysis of current drinkers, consumption of champagne or white wine, beer or cider, and spirits each was associated with a higher risk of gout among both sexes, with the strongest association observed for beer or cider, which may be due to the higher level of purine in these alcoholic beverages.^6^

We initially observed, both among male and female drinkers, that light to moderate consumption of several specific alcoholic beverages was associated with a lower risk of gout. After mutually adjusting for the specific alcoholic beverages and addressing potential reverse causation, we found that all of these associations were nullified. It is likely that some participants with illness or ill health had abstained from alcohol before being recruited to the study and self-identified as nondrinkers or occasional drinkers during the baseline assessment. Including such at-risk individuals can increase the risk within the nondrinking or occasional drinking group and lead to a U- or J-shaped association between alcohol consumption and health risk.^17,18^ Our findings and other previous findings on alcohol^17,18^ and nonalcohol lifestyle factors^16^ underscore the importance of considering reverse causation bias for a more accurate estimation of the assessed association with health outcomes.

### Limitations

The present study has several limitations. First, despite the careful consideration of potential confounders and reverse causation bias, residual confounding cannot be fully excluded. Second, although the UK Biobank is a prospective cohort study, the present study was not specifically designed before the recruitment of the participants. Third, because the frequency of alcohol consumption was self-reported, some degree of misclassification of exposure was inevitable. Fourth, alcohol consumption was assessed only at baseline. A recent study from Japan^34^ found that reducing alcohol intake (especially beer) or complete discontinuation of alcohol (especially for hyperuricemic individuals) was associated with longitudinal decreases in serum urate levels. Fifth, the intake of fortified wine was relatively low in this population, such that the analysis is likely underpowered to detect a moderate association. Sixth, incident cases of gout were identified from hospital records, such that there were likely some undiagnosed cases or gout diagnoses made only in primary care settings that were not identified. Seventh, most participants in the UK Biobank are of European descent who are White and are relatively healthier than the general population in the UK.^35^ Thus, further verification of the findings for other regional and racial and ethnic populations is needed.

## Conclusions

In this prospective cohort study with a careful consideration of potential confounding and reverse causation, consumption of several specific alcoholic beverages was associated with a higher risk of gout among both sexes. The observed sex-specific difference in the association of total alcohol consumption with incident gout may be owing to differences between men and women in the types of alcohol consumed rather than biological differences.

## Notes

Supplement 1. **eTable 1.** Sex-Specific Baseline Participant Characteristics According to Alcohol Drinking Status **eTable 2.** Sex-Specific Baseline Participant Characteristics According to Drinking Frequency Among Current Drinkers **eTable 3.** Sex-Specific Association Between Total Alcohol Consumption and Incident Gout Among Current Drinkers **eFigure 1.** Kaplan-Meier Curves for Incident Gout Over Categories of Total Alcohol Consumption Among Current Drinkers **eFigure 2.** Spearman Partial Correlations Among Specific Alcoholic Beverages Among Current Drinkers **eFigure 3.** Pure Ethanol (per 8 g/d) From Total or Specific Alcoholic Beverages and Incident Gout Among Current Drinkers

Supplement 2.
Data Sharing Statement
