# Supplementary material for: Consumption of Total and Specific Alcoholic Beverages and Long-Term Risk of Gout Among Men and Women
Source: JAMA Netw Open. 2024 Aug 28;7(8):e2430700. doi: 10.1001/jamanetworkopen.2024.30700 (PMC11358860; doi:10.1001/jamanetworkopen.2024.30700)
Supplement: Supplement 1. — eTable 1. Sex-Specific Baseline Participant Characteristics According to Alcohol Drinking Status eTable 2. Sex-Specific Baseline Participant Characteristics According to Drinking Frequency Among Current Drinkers eTable 3. Sex-Specific Association Between Total Alcohol Consumption and Incident Gout Among Current Drinkers eFigure 1. Kaplan-Meier Curves for Incident Gout Over Categories of Total Alcohol Consumption Among Current Drinkers eFigure 2. Spearman Partial Correlations Among Specific Alcoholic Beverages Among Current Drinkers eFigure 3. Pure Ethanol (per 8 g/d) From Total or Specific Alcoholic Beverages and Incident Gout Among Current Drinkers [file jamanetwopen-e2430700-s001.pdf]

Supplementary Online Content

Lyu JQ, Miao MY, Wang JM, et al. Consumption of total and specific alcoholic beverages and long-term risk of gout among men and women. *JAMA Netw Open.* 2024;7(8):e2430700. doi:10.1001/jamanetworkopen.2024.30700

eTable 1. Sex-Specific Baseline Participant Characteristics According to Alcohol Drinking Status

eTable 2. Sex-Specific Baseline Participant Characteristics According to Drinking Frequency Among Current Drinkers

eTable 3. Sex-Specific Association Between Total Alcohol Consumption and Incident Gout Among Current Drinkers

eFigure 1. Kaplan-Meier Curves for Incident Gout Over Categories of Total Alcohol Consumption Among Current Drinkers

eFigure 2. Spearman Partial Correlations Among Specific Alcoholic Beverages Among Current Drinkers

eFigure 3. Pure Ethanol (per 8 g/d) From Total or Specific Alcoholic Beverages and Incident Gout Among Current Drinkers

This supplementary material has been provided by the authors to give readers additional information about their work.

© 2024 Lyu JQ et al. *JAMA Network Open.*

eTable 1. Sex-Specific Baseline Participant Characteristics According to Alcohol Drinking Status

Characteristics	Men, No. (%)			Women, No. (%)		
	Never Drinking (n = 6253)	Former drinking (n = 7792)	Current drinking (n = 203 919)	Never drinking (n = 15 879)	Former drinking (n = 9881)	Current drinking (n = 245 497)
**Age, mean (SD), y**	55.48 (9.1)	56.88 (8.1)	56.63 (8.2)	57.39 (8.3)	57.29 (7.8)	56.22 (8.0)
**Ethnicity**						
Asian or Asian British	39 (0.7)	56 (0.7)	939 (0.5)	143 (1.0)	88 (1.0)	1575 (0.7)
Black or Black British	1505 (26.4)	383 (5.1)	3108 (1.6)	2313 (15.6)	134 (1.34)	2057 (0.9)
White	3700 (64.9)	6864 (91.2)	194 340 (96.7)	11 451 (77.4)	9268 (95.4)	235 045 (97.1)
Other[a]	455 (8.0)	220 (2.9)	2623 (1.3)	888 (6.0)	223 (2.3)	3474 (1.43)
**TDI, mean (SD)**	0.27 (3.6)	0.33 (3.6)	-1.37 (3.1)	-0.35 (3.4)	-0.30 (3.4)	-1.45 (3.0)
**Smoking status,**						
Never	4795 (77.3)	2960 (38.2)	99 332 (48.9)	13 167 (83.4)	4869 (49.6)	143 139 (58.5)
Former	854 (13.8)	3384 (43.7)	78 218 (38.5)	1742 (11.0)	3563 (36.3)	79 603 (32.5)
Current: <10 pack-years	85 (1.4)	130 (1.7)	2890 (1.4)	85 (0.6)	132 (1.3)	3212 (1.3)
Current: 10-50 pack-years	423 (6.8)	1011 (13.1)	20 168 (9.9)	720 (4.6)	1132 (11.5)	17 631 (7.2)
Current: >50 pack-years	44 (0.7)	257 (3.3)	2570 (1.3)	76 (0.5)	126 (1.3)	1081 (0.4)
**Physical activity, MET-h/week, mean (SD)**	40.43 (45.3)	44.40 (49.3)	44.34 (45.3)	38.29 (38.9)	39.56 (39.6)	38.89 (36.4)
**Average dietary intake, mean (SD)**						
Red meat, servings/week	2.1 (1.9)	2.1 (1.8)	2.3 (1.5)	1.8 (1.7)	1.7 (1.4)	2.0 (1.4)
Processed meat, servings/week	1.6 (1.7)	1.7 (1.7)	1.9 (1.5)	1.1 (1.3)	1.1 (1.2)	1.2 (1.2)
Poultry, servings/week	1.9 (1.5)	1.8 (1.4)	1.9 (1.2)	1.8 (1.4)	1.8 (1.4)	1.9 (1.3)
Oily fish, servings/week	1.0 (1.3)	1.0 (1.2)	1.1 (1.1)	1.0 (1.2)	1.1 (1.2)	1.1 (1.0)
Non-oily fish, servings/week	1.1 (1.1)	1.1 (1.1)	1.1 (0.9)	1.1 (1.1)	1.1 (1.0)	1.2 (0.9)
Fresh fruit, pieces/week	16.5 (16.2)	16.5 (14.3)	14.1 (11.0)	18.4 (13.9)	18.4 (13.6)	16.6 (10.8)
Fresh vegetables, tablespoons/week	16.1 (22.1)	13.8 (17.2)	13.6 (14.5)	16.7 (17.0)	16.6 (17.3)	16.6 (14.8)
**Hypertension**	3643 (58.3)	4827 (62.0)	128 289 (62.9)	8845 (55.7)	5 164 (52.3)	120 692 (49.1)
**Dyslipidemia**	1760 (28.2)	2424 (31.1)	48 105 (23.6)	3520 (22.2)	2099 (21.2)	32 352 (13.2)
**Diabetes**	999 (16.0)	1187 (15.2)	14 577 (7.2)	1672 (10.5)	890 (9.0)	9218 (3.8)
**eGFR, mL/min/1.73 m^2, mean (SD)**	91.16 (14.5)	91.19 (14.3)	90.84 (12.5)	90.44 (14.3)	90.51 (13.8)	91.29 (12.7)
**Diuretics use**	114 (1.8)	257 (3.3)	2662 (1.3)	306 (1.9)	252 (2.6)	2482 (1.0)
**BMI, kg/m^2, mean (SD)**	27.73 (4.7)	28.18 (5.0)	27.70 (4.1)	28.25 (5.9)	28.23 (6.1)	26.94 (5.1)

Abbreviations: BMI, body mass index; TDI, Townsend deprivation index; MET, metabolic equivalent.

[a] Other was defined as any other race or ethnicity not otherwise specified

© 2024 Lyu JQ et al. *JAMA Network Open.*

eTable 2. Sex-Specific Baseline Participant Characteristics According to Drinking Frequency Among Current Drinkers

Characteristics	Men, No. (%)				Women, No. (%)			
	<1/week (n = 36 110)	1-2/week (n = 56 773)	3-4/week (n = 56 672)	≥5/week (n = 54 364)	<1/week (n = 76 249)	1-2/week (n = 69 881)	3-4/week (n = 55 693)	≥5/week (n = 43 674)
**Age, mean (SD), y**	55.85 (8.6)	55.88 (8.4)	56.53 (8.0)	58.06 (7.7)	56.27 (8.1)	55.77 (8.0)	55.82 (7.8)	57.39 (7.6)
**Ethnicity, %**								
Asian or Asian British	263 (0.8)	273 (0.5)	204 (0.4)	199 (0.4)	683 (0.9)	402 (0.6)	270 (0.5)	220 (0.5)
Black or Black British	1163 (3.3)	873 (1.6)	576 (1.0)	496 (0.9)	1307 (1.8)	418 (0.6)	201 (0.4)	131 (0.3)
White	32 539 (92.9)	54 062 (96.5)	54 902 (97.8)	52 837 (98.1)	70 137 (94.4)	67 574 (97.7)	54 509 (98.6)	42 825 (98.8)
Other[a]	1078 (3.1)	802 (1.4)	434 (0.8)	309 (0.6)	2207 (3.0)	758 (1.1)	326 (0.6)	183 (0.4)
**Townsend deprivation index**	-0.71 (3.4)	-1.31 (3.1)	-1.68 (2.9)	-1.55 (3.0)	-0.91 (3.2)	-1.55 (2.9)	-1.84 (2.7)	-1.71 (2.8)
**Smoking status,**								
Never	19 709 (54.9)	30 692 (54.3)	28 400 (50.3)	20 531 (37.9)	48 302 (63.6)	43 473 (62.4)	31 624 (57.0)	19 740 (45.3)
Former	11 049 (30.8)	19 478 (34.4)	22 332 (39.5)	25 359 (46.8)	20 109 (26.5)	20 705 (29.7)	19 883 (35.8)	18 906 (43.4)
Current: <10 pack-years	510 (1.42)	651 (1.15)	678 (1.2)	1051 (1.94)	897 (1.18)	763 (1.1)	708 (1.28)	844 (1.94)
Current: 10-50 pack-years	4057 (11.29)	5210 (9.21)	4650 (8.23)	6251 (11.53)	6237 (8.21)	4486 (6.44)	3161 (5.69)	3747 (8.61)
Current: >50 pack-years	594 (1.65)	522 (0.92)	443 (0.78)	1011 (1.87)	421 (0.55)	213 (0.31)	148 (0.27)	299 (0.69)
**Physical activity, MET-h/week, mean (SD)**	45.01 (48.1)	45.72 (46.6)	43.47 (43.0)	43.36 (44.5)	38.77 (37.7)	38.77 (35.8)	38.68 (35.0)	39.53 (36.7)
**Average dietary intake**								
Red meat, servings/week	2.1 (1.6)	2.2 (1.4)	2.3 (1.5)	2.4 (1.6)	1.9 (1.4)	2.0 (1.3)	2.0 (1.3)	2.1 (1.4)
Processed meat, servings/week	1.8 (1.6)	1.9 (1.5)	1.9 (1.5)	2.0 (1.6)	1.1 (1.2)	1.2 (1.2)	1.2 (1.2)	1.2 (1.2)
Poultry, servings/week	1.9 (1.3)	1.9 (1.2)	2.0 (1.2)	1.9 (1.2)	1.9 (1.3)	2.0 (1.2)	1.9 (1.2)	1.9 (1.2)
Oily fish, servings/week	1.0 (1.1)	1.1 (1.0)	1.1 (1.1)	1.2 (1.1)	1.1 (1.0)	1.1 (1.0)	1.2 (1.0)	1.2 (1.0)
Non-oily fish, servings/week	1.1 (1.0)	1.1 (0.9)	1.2 (0.9)	1.2 (0.9)	1.1 (1.0)	1.2 (0.9)	1.2 (0.9)	1.2 (1.0)
Fresh fruit, pieces/week	15.0 (12.4)	14.8 (11.4)	14.3 (10.1)	12.6 (10.3)	17.3 (11.6)	17.0 (10.8)	16.4 (10)	15 (10.3)
Fresh vegetables, tablespoons/week	13.4 (16.8)	13.6 (14.5)	13.8 (13.6)	13.7 (13.9)	16 (15.6)	16.5 (14.5)	17.1 (14.1)	17.3 (14.9)
**Hypertension**	21 926 (60.7)	34 315 (60.4)	35 434 (62.5)	36 614 (67.4)	38 802 (50.9)	33 144 (47.4)	26 234 (47.1)	22 512 (51.6)
**Dyslipidemia**	9336 (25.9)	12 857 (22.7)	12 645 (22.3)	13 267 (24.4)	12 605 (16.5)	8690 (12.4)	5947 (10.7)	5110 (11.7)
**Diabetes**	4121 (11.4)	4310 (7.6)	3106 (5.5)	3040 (5.6)	4828 (6.3)	2276 (3.3)	1195 (2.2)	919 (2.1)
**eGFR, mL/min/1.73 m^2, mean (SD)**	90.63 (13.7)	90.85 (12.7)	90.93 (12.1)	90.87 (12.0)	90.83 (13.5)	91.41 (12.7)	91.87 (12.2)	91.12 (12.1)
**Diuretics use**	662 (1.8)	703 (1.2)	625 (1.1)	672 (1.2)	1110 (1.5)	608 (0.9)	416 (0.8)	348 (0.8)
**BMI, kg/m^2**	28.28 (4.8)	27.97 (4.2)	27.51 (3.8)	27.24 (3.8)	28.19 (5.8)	26.97 (4.9)	26.15 (4.4)	25.74 (4.2)
**Average consumption of specific alcoholic beverages**								
Red wine, glasses/week	0.1 (0.2)	1.6 (2.3)	4.0 (4.1)	5.9 (5.6)	0.1 (0.2)	1.6 (2.0)	3.4 (3.3)	4.3 (4.3)
Champagne/white wine,	0.1 (0.2)	0.8 (1.5)	1.7 (2.6)	2.4 (3.5)	0.7 (0.2)	1.6 (2.0)	3.1 (3.4)	4.1 (4.4)

© 2024 Lyu J-Q et al. *JAMA Network Open.*

Characteristics	Men, No. (%)				Women, No. (%)			
	**<1/week** (n = 36 110)	**1-2/week** (n = 56 773)	**3-4/week** (n = 56 672)	**≥5/week** (n = 54 364)	**<1/week** (n = 76 249)	**1-2/week** (n = 69 881)	**3-4/week** (n = 55 693)	**≥5/week** (n = 43 674)
glasses/week								
Beer/cider, pints/week	0.2 (0.2)	3.2 (3.3)	4.8 (4.8)	5.3 (5.8)	0.1 (0.2)	0.5 (1.1)	0.5 (1.2)	0.51 (1.3)
Spirit, measures/week	0.8 (0.2)	1.0 (2.4)	1.6 (3.3)	2.8 (5.2)	0.8 (0.2)	0.9 (1.9)	1.2 (2.4)	1.56 (3.1)
Fortified wine, glasses/week	0.0 (0.1)	0.1 (0.5)	0.2 (0.7)	0.2 (0.9)	0.0 (0.1)	0.2 (0.6)	0.3 (0.8)	0.32 (1.1)

Abbreviations: BMI, body mass index; TDI, Townsend deprivation index; MET, metabolic equivalent.

[a] Other was defined as any other race or ethnicity not otherwise specified.

© 2024 Lyu J-Q et al. *JAMA Network Open.*

eTable 3. Sex-Specific Association Between Total Alcohol Consumption and Incident Gout Among Current Drinkers

Model	Drinking status, HR (95%CI)		
	Never	Former	Current
**Men**			
Case/Total	125/6253	196/7792	6240/203 919
Model 1	1.00 (Referent)	0.99 (0.78-1.26)	0.91 (0.78-1.07)
Model 2	1.00 (Referent)	1.18 (0.94-1.49)	1.57 (1.31-1.89)
Model 2 [a]	1.00 (Referent)	1.18 (0.94-1.48)	1.57 (1.30-1.88)
Model 2 [b]	1.00 (Referent)	1.10 (0.85-1.42)	1.58 (1.29-1.93)
Model 2 [c]	1.00 (Referent)	1.25 (0.94-1.65)	1.69 (1.35-2.12)
Model 2 [d]	1.00 (Referent)	1.24 (0.97-1.59)	1.60 (1.31-1.96)
Model 2 [e]	1.00 (Referent)	1.13 (0.81-1.57)	1.69 (1.30-2.18)
**Women**			
Case/Total	192/15 879	114/9881	1772/245 497
Model 1	1.00 (Referent)	1.03 (0.81-1.31)	0.85 (0.72-0.99)
Model 2	1.00 (Referent)	0.99 (0.78-1.26)	0.91 (0.78-1.07)
Model 2 [a]	1.00 (Referent)	0.99 (0.78-1.26)	0.90 (0.77-1.06)
Model 2 [b]	1.00 (Referent)	0.93 (0.70-1.22)	0.94 (0.79-1.12)
Model 2 [c]	1.00 (Referent)	0.91 (0.67-1.22)	0.87 (0.72-1.06)
Model 2 [d]	1.00 (Referent)	0.96 (0.75-1.24)	0.88 (0.74-1.05)
Model 2 [e]	1.00 (Referent)	0.85 (0.60-1.20)	0.83 (0.67-1.03)

Abbreviation: HR, hazard ratio.

Model 1 was adjusted for age (y), ethnic group (Asian/Asian British, Black/Black British, White, other), Townsend deprivation index, smoking (never, former, current [<10, 10-<50, >50 pack-years]), total physical activity (MET-h/week), hypertension (yes, no), dyslipidemia (yes, no), diabetes (yes, no), eGFR, diuretics use (yes, no), and major food groups (red meat, processed meat, poultry, oily fish, non-oily fish, fresh fruit, and fresh vegetables).

Model 2 was additionally adjusted for BMI (kg/m^2).

[a] Excluding current drinkers who had reduced alcohol intake for illness or ill health at baseline.

[b] Excluding participants self-rating as having poor health at baseline.

[c] Excluding participants with major cardiovascular disease, cancer, or renal failure at baseline.

[d] Excluding cases occurring within the first 2 years of follow-up.

[e] Excluding all above. P for interaction between men and women <0.001 in the main analysis.

© 2024 Lyu J-Q et al. *JAMA Network Open.*

Men

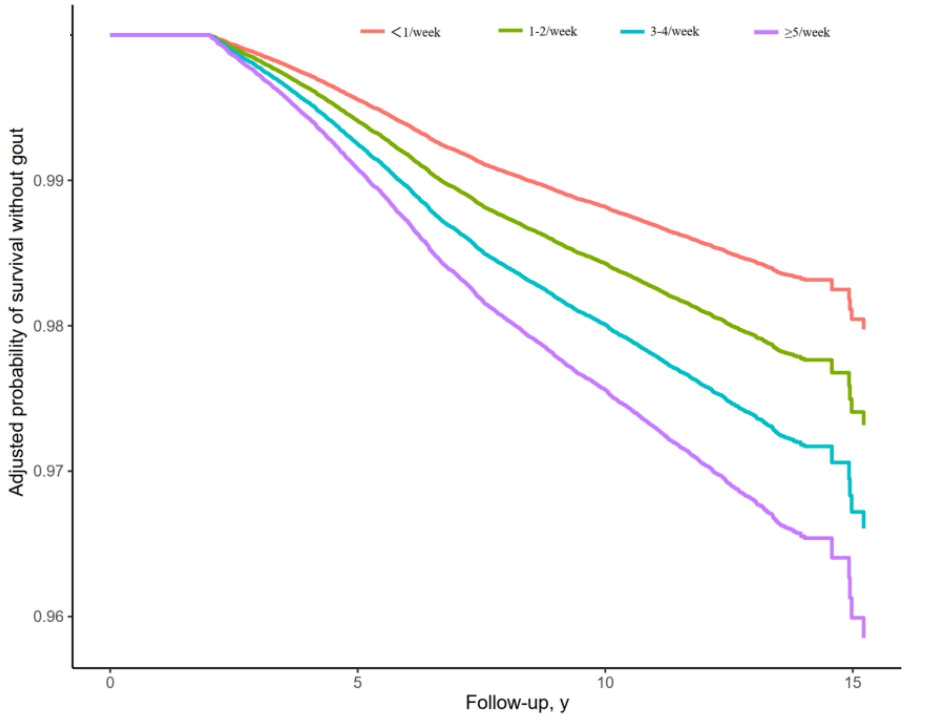

Women

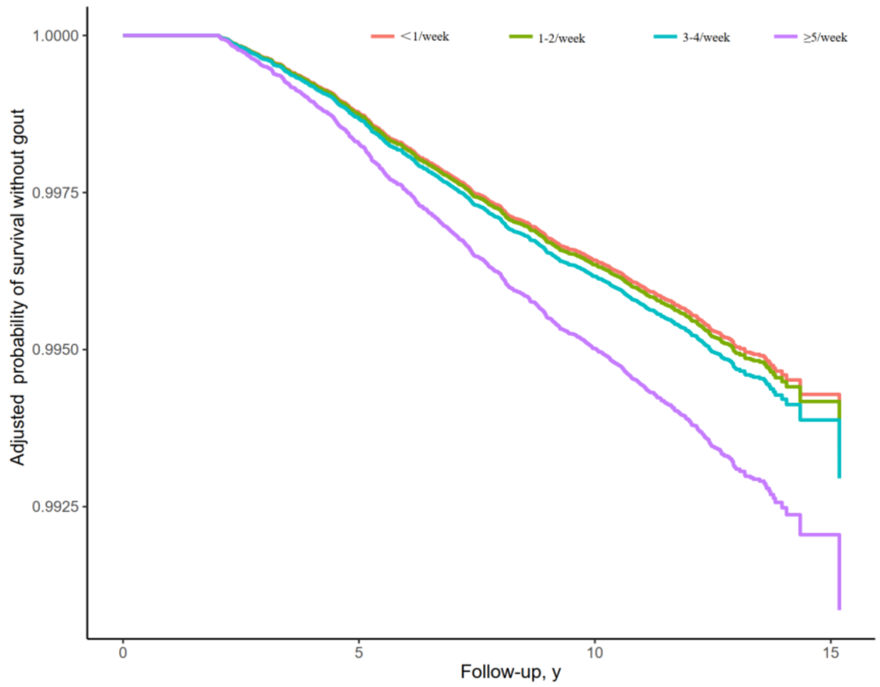

[a] Curves are adjusted for age (y), ethnic group (Asian/Asian British, Black/Black British, White, other), Townsend deprivation index, smoking (never, former, current [<10, 10-<50, >50 pack-years]), total physical activity (MET-h/week), hypertension (yes, no), dyslipidemia (yes, no), diabetes (yes, no), eGFR, diuretics use (yes, no), and major food groups (red meat, processed meat, poultry, oily fish, non-oily fish, fresh fruit, and fresh vegetables), and BMI.

[b] Excluded participants who had reduced alcohol intake for illness or ill health, self-rated as having poor health, or had major cardiovascular disease, cancer, or renal failure at baseline, in addition to incident cases occurring within the first 2 years of follow-up.

eFigure 1. Kaplan-Meier Curves for Incident Gout Over Categories of Total Alcohol Consumption Among Current Drinkers

© 2024 Lyu J-Q et al. *JAMA Network Open.*

Men

	Red wine	Champagne/white wine	Beer/cider	Spirits	Fortified wine
**Red wine**	1.00	0.14	-0.10	0.05	0.03
**Champagne/white wine**	0.14	1.00	-0.08	0.02	0.05
**Beer/cider**	-0.10	-0.08	1.00	0.03	-0.04
**Spirits**	0.05	0.02	0.03	1.00	0.04
**Fortified wine**	0.03	0.05	-0.04	0.04	1.00

Women

	Red wine	Champagne/white wine	Beer/cider	Spirits	Fortified wine
**Red wine**	1.00	-0.07	-0.04	-0.02	0.00
**Champagne/white wine**	-0.07	1.00	-0.05	-0.02	-0.01
**Beer/cider**	-0.04	-0.05	1.00	0.01	-0.01
**Spirits**	-0.02	-0.02	0.01	1.00	0.01
**Fortified wine**	0.00	-0.01	-0.01	0.01	1.00

eFigure 2. Spearman Partial Correlations Among Specific Alcoholic Beverages Among Current Drinkers
Results were adjusted for age (y), ethnic group (Asian/Asian British, Black/Black British, White, other), and Townsend deprivation index.

© 2024 Lyu J-Q et al. *JAMA Network Open.*

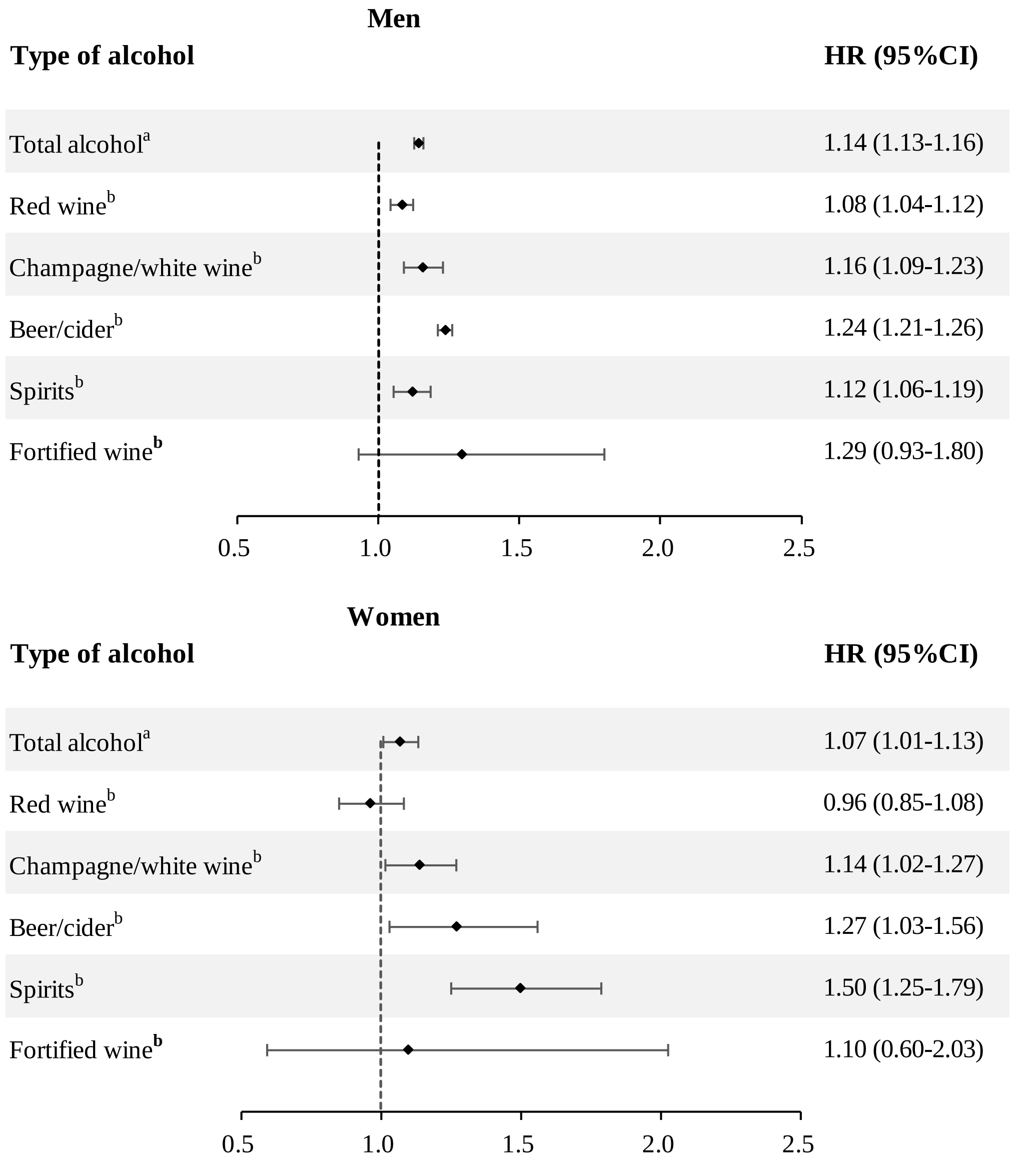

eFigure 3. Pure Ethanol (per 8 g/d) From Total or Specific Alcoholic Beverages and Incident Gout Among Current Drinkers
According to Lourida et al. (JAMA 2019;322:430-437), to estimate the amount of daily ethanol intake (g/d), the intake of each specific alcoholic beverages was first multiplied by a specific coefficient (0.85 for red wine and champagne/white wine, 1.28 for beer/cider, and 0.57 for spirits and fortified wine) to derive drink equivalent. One drink-equivalent contains 14 g of pure ethanol. Total alcohol indicates ethanol from all the listed specific alcoholic beverages.

[a] Results for total alcohol were adjusted for age (y), ethnic group (Asian/Asian British, Black/Black British, White, other), Townsend deprivation index, smoking (never, former, current [<10, 10-<50, >50 pack-years]), total physical activity (MET-h/week), hypertension (yes, no), dyslipidemia (yes, no), diabetes (yes, no), eGFR, diuretics use (yes, no), major food groups (red meat, processed meat, poultry, oily fish, non-oily fish, fresh fruit, and fresh vegetables), and BMI (kg/m^2).
[b] Results for specific alcohol were additionally adjusted for other types of alcoholic beverages.

All analyses excluded participants who had reduced alcohol intake for illness or ill health, self-rated as having poor health, or had major cardiovascular disease, cancer, or renal failure at baseline, in addition to incident cases occurring within the first 2 years of follow-up.

© 2024 Lyu J-Q et al. *JAMA Network Open.*
