# Supplementary material for: Consumption of Total and Specific Alcoholic Beverages and Long-Term Risk of Gout Among Men and Women
Source: JAMA Netw Open. 2024 Aug 28;7(8):e2430700. doi: 10.1001/jamanetworkopen.2024.30700 (PMC11358860; doi:10.1001/jamanetworkopen.2024.30700)
Supplement: Supplement 2. — Data Sharing Statement [file jamanetwopen-e2430700-s002.pdf]

Data Sharing Statement

Lyu. Consumption of Total and Specific Alcoholic Beverages and Long-Term Risk of Gout Among Men and Women. *JAMA Netw Open*. Published August 28, 2024. doi:10.1001/jamanetworkopen.2024.30700

Data
Data available: No
